# Establishing priorities for psychological interventions in pediatric settings: A decision-tree approach using the DISABKIDS-10 Index as a screening instrument

**DOI:** 10.1371/journal.pone.0198402

**Published:** 2018-05-31

**Authors:** Neuza Silva, Monika Bullinger, Helena Moreira, Maria Cristina Canavarro, Carlos Carona

**Affiliations:** 1 Cognitive and Behavioral Center for Research and Intervention, Faculty of Psychology and Education Sciences of the University of Coimbra, Coimbra, Portugal; 2 Department of Medical Psychology, University Medical Center Hamburg-Eppendorf, Hamburg, Germany; 3 Federation of Portuguese Cerebral Palsy Associations, Cerebral Palsy Association of Coimbra, Coimbra, Portugal; University of Calgary, CANADA

## Abstract

Most children and adolescents with chronic health conditions have impaired health-related quality of life and are at high risk of internalizing and externalizing problems. However, few patients present clinically significant symptoms. Using a decision-tree approach, this study aimed to identify risk profiles for psychological problems based on measures that can be easily scored and interpreted by healthcare professionals in pediatric settings. The participants were 736 children and adolescents between 8–18 years of age with asthma, epilepsy, cerebral palsy, type-1diabetes or obesity. The children and adolescents completed self-report measures of health-related quality of life (DISABKIDS-10) and psychological problems (Strengths and Difficulties Questionnaire). Sociodemographic and clinical data were collected from their parents/ physicians. Children and adolescents were classified into the normal (78.5%) or borderline/clinical range (21.5%) according to the Strengths and Difficulties Questionnaire cut-off values for psychological problems. The overall accuracy of the decision-tree model was 78.1% (sensitivity = 71.5%; specificity = 79.9%), with 4 profiles predicting 71.5% of borderline/clinical cases. The strongest predictor of psychological problems was a health-related quality of life standardized score below the threshold of 57.5 for patients with cerebral palsy, epilepsy or obesity and below 70.0 for patients with asthma or diabetes. Other significant predictors were low socio-economic status, single-parent household, medication intake and younger age. The model showed adequate validity (risk = .28, SE = .02) and accuracy (area under the Receiver Operating Characteristic curve = .84; CI = .80/.87). The identification of pediatric patients at high risk for psychological problems may contribute to a more efficient allocation of health resources, particularly with regard to their referral to specialized psychological assessment and intervention.

## Introduction

Over the past few decades, scientific and technological advances in medicine have resulted in epidemiological changes marked by an increased prevalence of pediatric chronic health conditions (estimated prevalence rates between 15% and 18% [1,2]) and by the emergence of patients’ subjective perceptions of their physical, psychological and social functioning and well-being as the primary goals of clinical interventions [3]. Evidence-based research has demonstrated that children and adolescents with chronic conditions are at a greater risk for psychosocial impairments; however, few patients present clinically significant psychological symptoms [4–6]. In addition, the practice of routine psychological assessments for all pediatric patients is not feasible due to the lack of time and human and financial resources in healthcare services [7,8]. Self-report screening tools that can be easily and promptly administered, scored and interpreted by any healthcare provider (e.g., general practitioner or nurses), and not only by specialized mental health professionals, may represent a promising way to identify and refer at-risk children and adolescents for specialized psychological assessments and interventions, thus contributing to a more efficient allocation of health resources and to an improved patient satisfaction with the quality of received care [9].

The most widely accepted models of child adjustment to pediatric chronic health conditions conceptualize adaptation as a multi-dimensional construct, including physical health, mental health (not just psychological maladjustment) and social functioning [10]. Internalizing and externalizing problems have traditionally been assessed as specific psychological functioning outcomes because of their high prevalence in children with chronic conditions or disabilities [11]. More recently, health-related quality of life (HrQoL), defined as “a multidimensional construct covering physical, emotional, mental, social, and behavioral components of well-being and function as perceived by patients and/or other observers” ([12], p. 344), has increasingly been acknowledged as a key health outcome in epidemiological and clinical studies, extending beyond the psychopathological conceptualization of child mental health and representing adaptation as a process that accounted for resiliency and variability on specific indicators [13]. Pediatric chronic health conditions may impact both psychological problems and HrQoL and these two indicators of psychosocial adaptation are expected to influence each other reciprocally [14]. In fact, empirical research has shown lower levels of perceived social support [15] and poorer HrQoL in children/adolescents with chronic conditions compared to their healthy peers [3,16] and has found that HrQoL impairments significantly account for more psychological problems [17]. Additional risk factors for psychological problems include the diagnosis of a medical condition that affects brain function, male gender, younger age, low socioeconomic status (SES), single-parent household, and parents’ mental health problems [5,18,19].

In primary care practice, physicians tend to underestimate psychosocial and functional impairments, and even severe psychological problems are often undetected and untreated, mostly because of lack of time and expertise in psychological assessment [20–22]. Two randomized controlled trials have demonstrated that providing patient-reported HrQoL information to primary care physicians improved patient-physician communication and increased detection and referral to mental health specialists [23,24]. However, pediatric HrQoL measures are far from being used routinely in clinical practice, despite the recognition of their utility to facilitate patient-physician communication, to improve patient satisfaction with the quality of medical care, to estimate the healthcare needs of specific populations, to longitudinally monitor the disease status and effectiveness of treatment, to detect psychological problems secondary to medical conditions or treatments (so-called “hidden morbidities”), and to assist physicians in clinical decision-making and referral processes [25,26]. In addition, HrQoL scores can be difficult to interpret in clinical practice because they are often presented as the mean values along the continuum from excellent to poor health [25,27], and population-based clinically meaningful cut-off points to identify at-risk patients are unavailable for most HrQoL instruments.

In this context of routine monitoring and screening, short-form questionnaires that summarize scores into a single value (or index) have been found to be reliable and valid measures for global HrQoL assessment, while reducing response burden and saving administration costs [28,29]. The DISABKIDS-10 Index [30] is a commendable example of a short-form measure specifically designed to assess the perceived impact of chronic health conditions on children’s and adolescents’ physical, mental and social well-being and functioning. This chronic-generic instrument was built upon a non-categorical approach, which suggests that nosologically different health conditions may lead to similar impacts on patients’ HrQoL [31,32], thus allowing for comparisons across different diagnosis without sacrificing the sensitivity to specific psychosocial impairments resulting from the health condition. In addition, the DISABKIDS questionnaires are child-centered and developmentally appropriate, use subjective self-report whenever possible (proxy judgments can be used if the child is too young or too disabled to complete self-reports), are cross-culturally comparable, and emphasize health-enhancing aspects of HrQoL rather than merely listing symptoms, as recommended by the WHO Division of Mental Health [33]. The current challenge is to maximize the use of HrQoL measures in pediatric healthcare services, by enhancing their interpretability as screening tools to detect psychosocial and functional disabilities secondary to the medical condition and to support the adequate referral to specialized mental healthcare. Thus, the current study aimed (1) to compare the levels of HrQoL and the prevalence of borderline/clinically significant psychological problems across pediatric patients with different chronic health conditions (i.e., asthma, epilepsy, cerebral palsy, type-1 diabetes, and overweight/obesity) and (2) to test a classification-tree model to identify risk profiles for the development of borderline/clinically significant psychological problems based on the DISABKIDS-10 Index as a HrQoL screening measure and on patients’ and families’ sociodemographic and clinical data.

## Methods

### Participants and procedures

The participants were children and adolescents with chronic health conditions who were recruited at the outpatient pediatric services of three Portuguese public hospitals and 10 Cerebral Palsy Associations. For inclusion in the sample, the children/adolescents had to meet the following criteria: (1) age between 8 and 18 years at the time of recruitment; (2) diagnosis of asthma, cerebral palsy, type-1 diabetes, epilepsy or overweight/obesity according to the International Classification of Diseases-10 [34]; (3) absence of other comorbid chronic health conditions; (4) ability to understand and answer self-report questionnaires; and (5) accompanied by the parent currently assuming the primary caregiver role. In addition, pediatric patients with asthma or epilepsy were required to have the diagnosis for at least one year, children and adolescents with cerebral palsy were eligible if they had a minimum intelligence quotient (IQ) of 70, and children and adolescents with overweight/obesity were included when their body mass index (BMI) was above the 85^th^ percentile for same age and same sex peers according to the Centers for Disease Control and Prevention growth curves [35]. These diagnoses were selected for inclusion in this study because they share non-nosological clinical features that may impact HrQoL and psychological functioning, such as central nervous system impairment (epilepsy and cerebral palsy), external visibility (obesity and cerebral palsy), unpredictability of crises/exacerbations (epilepsy and asthma), and need for treatments that require changes in daily routines (diabetes and obesity).

Data collection occurred between March 2009 and December 2012, after the study had been approved by the institutions’ Ethics Committees/Direction Boards. Using the non-probabilistic convenience sampling method, children and adolescents who attended medical routine appointments in the period of sample collection were screened by health professionals based on their medical records and those who met the aforementioned inclusion criteria were invited to participate in the study. The study’s aims and procedures were explained in detail, and written consent forms were obtained from all parents and adolescents older than 13 years; younger children provided verbal informal assent. Children and adolescents who agreed to participate completed the self-report questionnaires in the institution they attended under the supervision of a trained research assistant, and their parents were asked to complete a sociodemographic and clinical datasheet.

### Measures

#### Psychological problems

To assess the children’s and adolescents’ psychological problems, we used the Difficulties scale of the Portuguese self-rated version of the Strengths and Difficulties Questionnaire (SDQ) [36,37], which is a brief behavioral questionnaire that was developed with reference to the main nosological categories recognized by the Diagnostic and Statistical Manual of Mental Disorders, 4^th^ edition (DSM-IV) [38]. This scale comprises 20 items assessing emotional symptoms (e.g., “I am often unhappy, down-hearted or tearful”), conduct problems (e.g., “I fight a lot. I can make other people do what I want”), hyperactivity/inattention (e.g., “I am easily distracted, I find it difficult to concentrate”) and peer relationship problems (e.g., “Other children or young people pick on me or bully me”). The items were answered using a Likert-type response scale with three options (0 = *not true*, 1 = *somewhat true*, and 2 = *certainly true*), providing a Total Difficulties score, with higher values indicating more psychological problems. Adequate reliability was obtained for the Difficulties scale (α = .76). The Total Difficulties sum score was dichotomized based on published cut-off values [37] to differentiate between pediatric patients within the normal (0–15 points; *n* = 578) and the borderline/clinical range (16–40 points; *n* = 158).

#### Health-related quality of life

Children’s and adolescents’ HrQoL was assessed by the Portuguese self-report short version of the DISABKIDS-10 Index [30,39]. This short-form questionnaire contains 10 items measuring the physical, mental and social impact of chronic health conditions (e.g., “Does your condition get you down?”) on 8- to 18-year-old patients’ lives and is answered on a 5-point Likert scale ranging from 1 (*never*) to 5 (*always*). According to its one-dimensional factor structure, a standardized composite score (0–100), representing the physical, mental and social domains of HrQoL, was calculated, with higher scores indicating better chronic-generic HrQoL. In the current sample, the questionnaire presented good reliability, with a Cronbach’s α value of .84.

#### Sociodemographic and clinical data

Sociodemographic data were reported by the parents and included their children’s age and gender, as well as parents’ education level, occupation, family structure and psychiatric history. Using a classification system specifically developed for the Portuguese context and based on the educational level and current job of the primary caregiver [40], the family SES was classified into low (e.g., unqualified employees in construction or manufacturing without completing the 9^th^ grade of school education), medium (e.g., employees in bureaus or banks, nurses, or teachers with intermediate or university courses) and high (e.g., senior officials of government, army, commerce or industry, physicians, or engineers with bachelor’s, master’s, doctorate or other post-graduate degrees). Due to the heterogeneous distribution of the SES levels observed in our sample, this variable was dichotomized into low (*n* = 464) and medium/high (*n* = 272).

Clinical data were provided by the parents and/or physicians and included the use of medication and specific information for each clinical group. Specifically, the physicians classified asthma severity into 4 levels (*intermittent*, *mild persistent*, *moderate persistent* and *severe persistent*) according to the Global Initiative for Asthma guidelines [41], and epilepsy severity was classified into 7 levels (from *not at all severe* to *extremely severe*) using the Global Assessment of Severity of Epilepsy Scale [42]. Levels of function in patients with cerebral palsy were classified into 5 levels according to the Gross Motor Function Classification System [43]. Values of glycated hemoglobin (HbA1c) at the time of assessment were obtained for patients with diabetes. The weight and height of patients with obesity were obtained from the parents and/or nutritionists. These markers of disease severity were dichotomized into mild (including patients with intermittent asthma, not at all to somewhat severe epilepsy, cerebral patients with level I of functioning with no limitations in walking, patients with diabetes with HbA1c ≤ 8%, and overweight children; *n* = 333 [45.2%]) and moderate/severe (including children/adolescents with mild, moderate and severe persistent asthma, moderately to extremely severe epilepsy, patients with cerebral palsy with levels II to V with movement restriction, patients with diabetes with HbA1c > 8%, and obese children; *n* = 315 [42.8%]).

### Data analyses

The statistical analyses were conducted with the Statistical Package for the Social Sciences (SPSS v.20.0; IBM Corp., Armonk, NY). Except for sociodemographic and clinical variables, missing data that were random (Little’s Missing Completely at Random [MCAR] Tests: χ_(12)_^2^ = 16.84, *p* = .16 for the SDQ Emotional Symptoms scale; χ_(12)_^2^ = 12.29, *p* = .42 for the SDQ Conduct Problems scale; χ_(8)_^2^ = 9.28, *p* = .32 for the SDQ Hyperactivity/Inattention scale; χ_(12)_^2^ = 5.14, *p* = .95 for the SDQ Peer Relationship Problems scale; and χ_(54)_^2^ = 39.68, *p* = .93 for the DISABKIDS-10 Index) and less than 5% of the values were replaced with the individual mean score for each variable. Descriptive statistics were obtained for sociodemographic and clinical variables and the sample characteristics were compared between diagnostic groups by using one-way analyses of variance (ANOVA) followed by post-hoc pairwise comparisons with Bonferroni correction for continuous variables and by χ^2^-tests for categorical variables.

The classification-tree model was performed using the Chi-Squared Automatic Interaction Detection (CHAID) algorithm, which is a classification method that also accounts for interactions between the predictors [44]. The CHAID method is based on chi-squared tests (χ^2^) that compare the squared deviations between observed and expected frequencies, with Bonferroni adjusted *p*-values for multiple comparisons with an overall error rate of .05 [45]. The algorithm starts by selecting the predictor that best discriminates the dependent variable (i.e., the predictor with the lowest *p*-value in the Chi-squared tests) and then splits the data set into two or more nodes (parent node). Subsequently, the method splits the new nodes into smaller nodes (child nodes) based on the variable that best discriminates each of them. The method ends when no more significant dependence relationships can be found between the dependent variable and the set of predictors. In the current study, nine eligible risk factors were included (age group, gender, diagnosis, HrQoL scores, medication, disease severity, SES, family structure and caregiver’s psychiatric history), and the target category was defined as having borderline/clinically significant psychological problems. The multicollinearity across the predictors was tested with preliminary correlation analysis and Variance Inflation Factor (VIF)/ Tolerance values. Because significant differences in most risk factors and the outcome variable were found between diagnostic groups, the health condition was forced into the model as the first split variable. Continuous independent variables were automatically recoded into discrete categories with minimal loss of information (interval = 10). The categories of each independent variable were merged if they were not significantly different with respect to the dependent variable; multiple branches were generated if the categories differed significantly (*p* ≤ .05). The minimum sample size for parent and child nodes were specified as *n* = 40 and *n* = 20, respectively, and the tree depth was limited to 4 levels. The likelihood ratio χ^2^-statistic was used because of the sample size < 1000 cases. The cost of misclassifying a child/adolescent with a high risk of psychological problems as low risk was customized as twice the cost of misclassifying a low risk patient as high risk.

To assess the predictive accuracy of the decision-tree model, the Receiver Operating Characteristic (ROC) curve was examined by plotting sensitivity (i.e., the proportion of patients with psychological problems who were correctly identified as positive) in the function of 1 –specificity (i.e., the proportion of patients without psychological problems who were correctly identified as negative) for different cut-off points of a parameter [44]. The statistic used to summarize the ROC analysis was the area under the curve (AUC), which corresponds to the probability that a randomly selected patient with borderline/clinical psychological symptoms was identified by the classification-tree model as higher risk than a randomly selected patient without psychological problems. AUC values between 0.90–1.00 were considered to have excellent accuracy, 0.80–0.90 were considered good, 0.70–0.80 were considered fair, 0.60–0.70 were considered poor, and 0.50–0.60 were considered failed [46,47].

## Results

### Descriptive statistics and analyses of variance

After excluding 115 cases due to comorbidities with other chronic conditions and 8 cases due to missing values in a ratio > 5% of the data, the final sample comprised 736 children/adolescents with asthma (*n* = 303), epilepsy (*n* = 98), cerebral palsy (*n* = 89), type-1 diabetes (*n* = 84) or overweight/obesity (*n* = 162). The sociodemographic and clinical characteristics for each diagnostic group are presented in Table 1.

**Table 1:** Sociodemographic and clinical characteristics of the sample (*N* = 736).

Patients’ characteristics		Asthma ^a^ (*n* = 303)	Epilepsy ^b^ (*n* = 98)	Cerebral Palsy ^c^ (*n* = 89)	Diabetes ^d^ (*n* = 84)	Obesity ^e^ (*n* = 162)	Differences between samples	
							*F*/χ^2^ (*df* = 4)	Pairwise comparisons
Age (years), *M* (*SD*)		12.31 (2.66)	11.96 (2.80)	12.06 (2.84)	13.06 (2.98)	13.02 (2.75)	4.15^**^	b < e
Age group, *n* (%)	Children 8-12y	166 (54.8%)	56 (57.1%)	47 (52.8%)	36 (42.9%)	70 (43.2%)	9.50^*^	a, b > e
	Adolescents 13-18y	137 (45.2%)	42 (42.9%)	42 (47.2%)	48 (57.1%)	92 (56.8%)		
Gender, *n* (%)	Male	186 (61.4%)	49 (50.0%)	49 (55.1%)	35 (41.7%)	69 (42.6%)	20.40^**^	a > d, e
	Female	117 (38.6%)	49 (50.0%)	40 (44.9%)	49 (58.3%)	93 (57.4%)		
Use of medication, *n* (%)		297 (98.0%)	86 (87.8%)	21 (23.6%)	84 (100.0%)	45 (27.8%)	410.80^**^	a, d > b, c, e; b > c, e
Disease severity, *n* (%)	Mild	166 (54.8%)	48 (49.0%)	56 (62.9%)	30 (35.7%)	33 (20.4%)	84.19^**^	a > b; a, b, c, d < e
	Moderate/severe	128 (42.2%)	17 (17.3%)	31 (34.8%)	15 (17.9%)	124 (76.5%)		
Psychological problems, *n* (%)	Borderline/clinical	50 (16.5%)	34 (34.7%)	21 (23.6%)	13 (15.5%)	40 (24.7%)	17.63^**^	b > a, d; e > a
	Normal	253 (83.5%)	64 (65.3%)	68 (76.4%)	71 (84.5%)	122 (75.3%)		
Health-related quality of life, *M* (*SD*)		85.17 (13.17)	81.84 (17.69)	76.88 (16.12)	82.47 (14.73)	80.66 (17.44)	5.89^**^	a > c, e
**Family characteristics**								
Socio-economic status, *n* (%)	Low	181 (59.7%)	66 (67.3%)	57 (64.0%)	46 (54.8%)	114 (70.4%)	8.45	-
	Medium/high	122 (40.3%)	32 (32.7%)	32 (36.0%)	38 (45.2%)	48 (29.6%)		
Family structure, *n* (%)	Single-parent	60 (19.8%)	26 (26.5%)	20 (22.5%)	6 (7.1%)	16 (9.9%)	21.03^**^	a, b, c > d, e
	Two-parents	243 (80.2%)	72 (73.5%)	69 (77.5%)	78 (92.9%)	146 (90.1%)		
Caregiver psychiatric history, *n* (%)		69 (22.8%)	22 (22.4%)	20 (22.5%)	24 (28.6%)	46 (28.4%)	3.15	-

^*^ *p* ≤ .05;

^**^ *p* ≤ .01, two-tailed.

The diagnostic groups differed significantly in terms of the patients’ age, gender and family structure. Bonferroni post-hoc analyses showed that patients with obesity were significantly older than patients with epilepsy, with a mean difference (*MD*) of 1.06 (*p* = .03). Pairwise comparisons with χ^2^-tests showed that the asthma group included significantly more boys compared to the diabetes [χ^2^_(1)_ = 10.44, *p* < .01] and obesity groups [χ^2^_(1)_ = 15.06, *p* < .01] and that children and adolescents with diabetes or obesity belonged to a single-parent household less often than did patients with asthma [χ^2^_(1)_ = 7.45, *p* < .01; χ^2^_(1)_ = 7.61, *p* < .01], epilepsy [χ^2^_(1)_ = 11.73, *p* < .01; χ^2^_(1)_ = 12.50, *p* < .01] or cerebral palsy [χ^2^_(1)_ = 7.95, *p* < .01; χ^2^_(1)_ = 7.42, *p* < .01]. No significant differences between diagnostic groups were found for family SES or the primary caregiver’s psychiatric history.

Regarding the clinical characteristics of the sample, patients with epilepsy were medicated less often than those with asthma [χ^2^_(1)_ = 18.20, *p* < .01] or diabetes were [χ^2^_(1)_ = 11.01, *p* < .01], whereas children and adolescents with cerebral palsy or obesity used medication less often than did patients with asthma [χ^2^_(1)_ = 248.82, *p* < .01; χ^2^_(1)_ = 267.72, *p* < .01], epilepsy [χ^2^_(1)_ = 78.44, *p* < .01; χ^2^_(1)_ = 87.87, *p* < .01] or diabetes [χ^2^_(1)_ = 105.74, *p* < .01; χ^2^_(1)_ = 115.69, *p* < .01]. In addition, pediatric asthma patients had better HrQoL than patients with cerebral palsy (*MD* = 8.29, *p* < .01) or obesity (*MD* = 4.51, *p* = .03) and fewer borderline/clinically significant psychological symptoms compared to children/adolescents with epilepsy [χ^2^_(1)_ = 14.80, *p* < .01] or obesity [χ^2^_(1)_ = 4.54, *p* = .04], whereas patients with diabetes had fewer psychological symptoms than the epilepsy group [χ^2^_(1)_ = 8.72, *p* < .01]. In terms of the specific clinical characteristics of the sample, more than half of pediatric asthma patients had intermittent asthma (*n* = 166, 54.8%); the mean severity score for patients with epilepsy was 2.63, *SD* = 1.31; the great majority of children and adolescents with cerebral palsy had a mild form of the condition, including spastic subtypes (*n* = 77, 91.7%) with no limitations in walking (*n* = 56, 62.9% at functional level I) and a mean IQ of 93.11, *SD* = 18.30 (range from 70 to 147); the mean glycated hemoglobin level of children with diabetes was 7.91, *SD* = 2.30%; and the standardized body mass index (zBMI) of children and adolescents with overweight/obesity was *M* = 1.90, *SD* = 0.41.

### Decision-tree model predicting borderline/clinical psychological symptoms

The preliminary analyses revealed weak to moderate correlations between the risk factors, with correlation coefficients ranging from .00 (between age group and disease severity) to -.55 (between diagnosis and medication). In addition the Variance Inflation Factor (VIF) values ranged from 1.03 to 1.78 (mean VIF = 1.08) and the Tolerance values were higher than .56, indicating that the model was not limited by multicollinearity problems.

**Fig 1:**
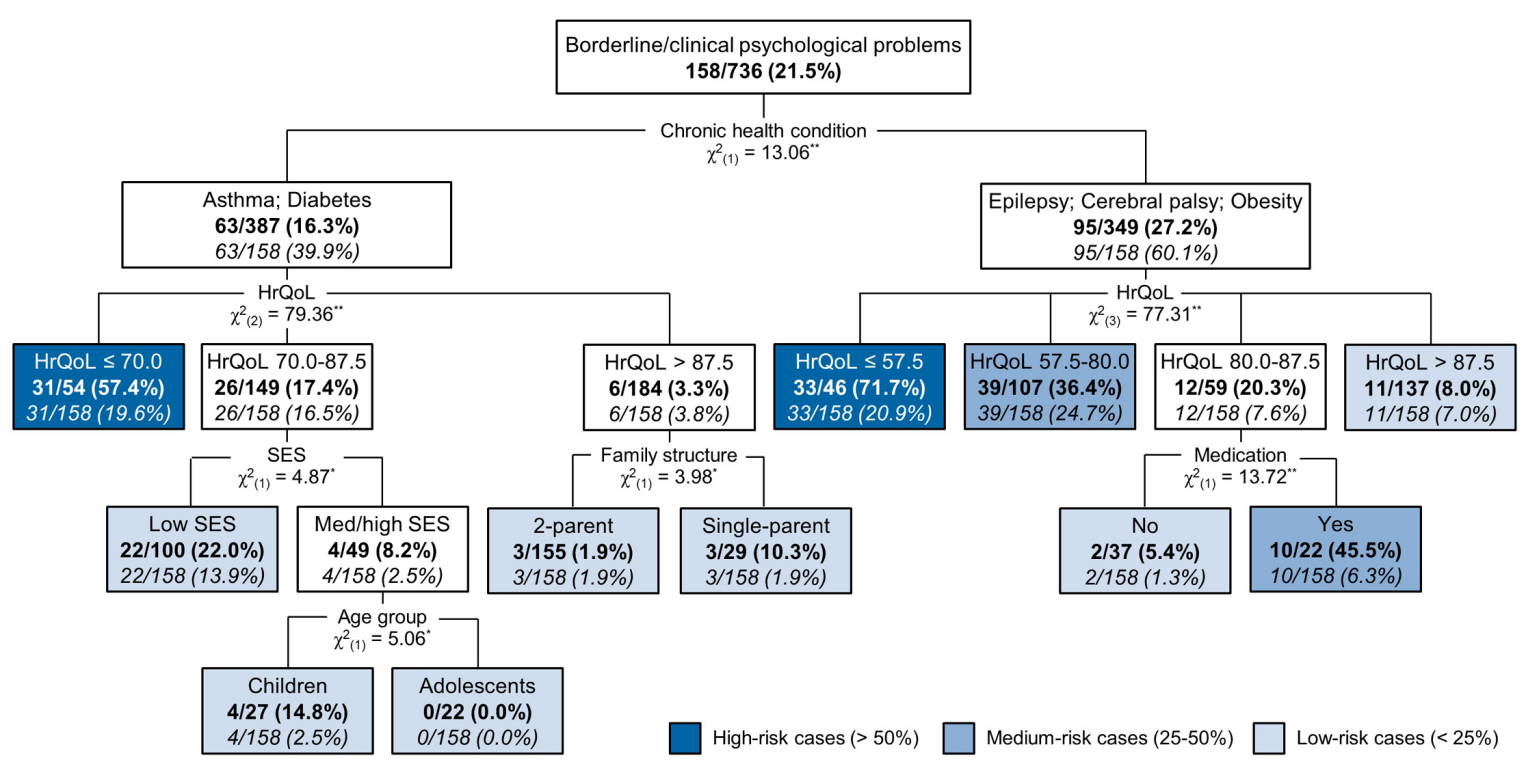
Decision-tree model predicting borderline/clinically significant psychological problems. *Note*. The colored terminal nodes show the rates of having borderline/clinical psychological symptoms within each profile (bold) and within the total target group (italic); The terminal nodes are colored coded into high-risk cases (> 50%); medium-risk cases (25–50%); and low-risk cases (< 25%); ^*^ *p* ≤ .05; ^**^ *p* ≤ .01, two-tailed.

The decision-tree model predicting borderline/clinical psychological symptoms is shown in Fig 1. The overall accuracy, i.e., the percentage of cases that were correctly predicted by the model was 78.1% (sensitivity = .72; specificity = .80; risk of misclassification = .28, *SE* = .02), with 11 terminal nodes derived from six predictors (diagnosis, HrQoL scores, family SES, family structure, age group, and use of medication). Children’s and adolescents’ gender, disease severity and caregiver’s psychiatric history were also included in the model as risk factors, but as they were non-significant predictors of psychological problems, they were automatically excluded from the model.

Beyond diagnosis, which was forced into the model as the first split variable, HrQoL was the most important predictor of psychological problems among pediatric patients, generating two high-risk profiles that predicted more than 50.0% of borderline/clinical cases: 71.7% of children and adolescents with cerebral palsy, epilepsy or obesity and HrQoL standardized scores below the threshold of 57.5 points and 57.4% of patients with asthma or diabetes and HrQoL scores below 70 points had borderline/clinically significant psychological symptoms. In addition, two medium-risk profiles were derived from the interaction between HrQoL scores and use of medication in pediatric patients with cerebral palsy, epilepsy or obesity: 36.4% of children/adolescents with HrQoL scores between 57.5–80 points and 45.5% of patients with HrQoL scores between 80–87.5 and using medication presented borderline/clinical psychological symptoms. Within the group with asthma or diabetes and HrQoL scores between 70–87.5, children and adolescents with low SES presented a greater likelihood of psychological problems (22.0%) compared to those with medium/high SES (8.2%); for patients with medium/high SES, the last significant predictor was age group, with younger children (14.8%) being at higher risk for psychological problems than adolescents (0.0%). For pediatric asthma or diabetes patients with HrQoL scores above 87.5, the last predictor was family structure, with children and adolescents living in a single-parent household being more likely to present psychological problems (10.3%) than their peers living in a two-parent family (1.9%). For patients with cerebral palsy, epilepsy and obesity and HrQoL scores above 87.5, no additional significant dependence relationships were found between the set of predictors and the outcome variable.

### ROC curve for the decision-tree model

**Fig 2:**
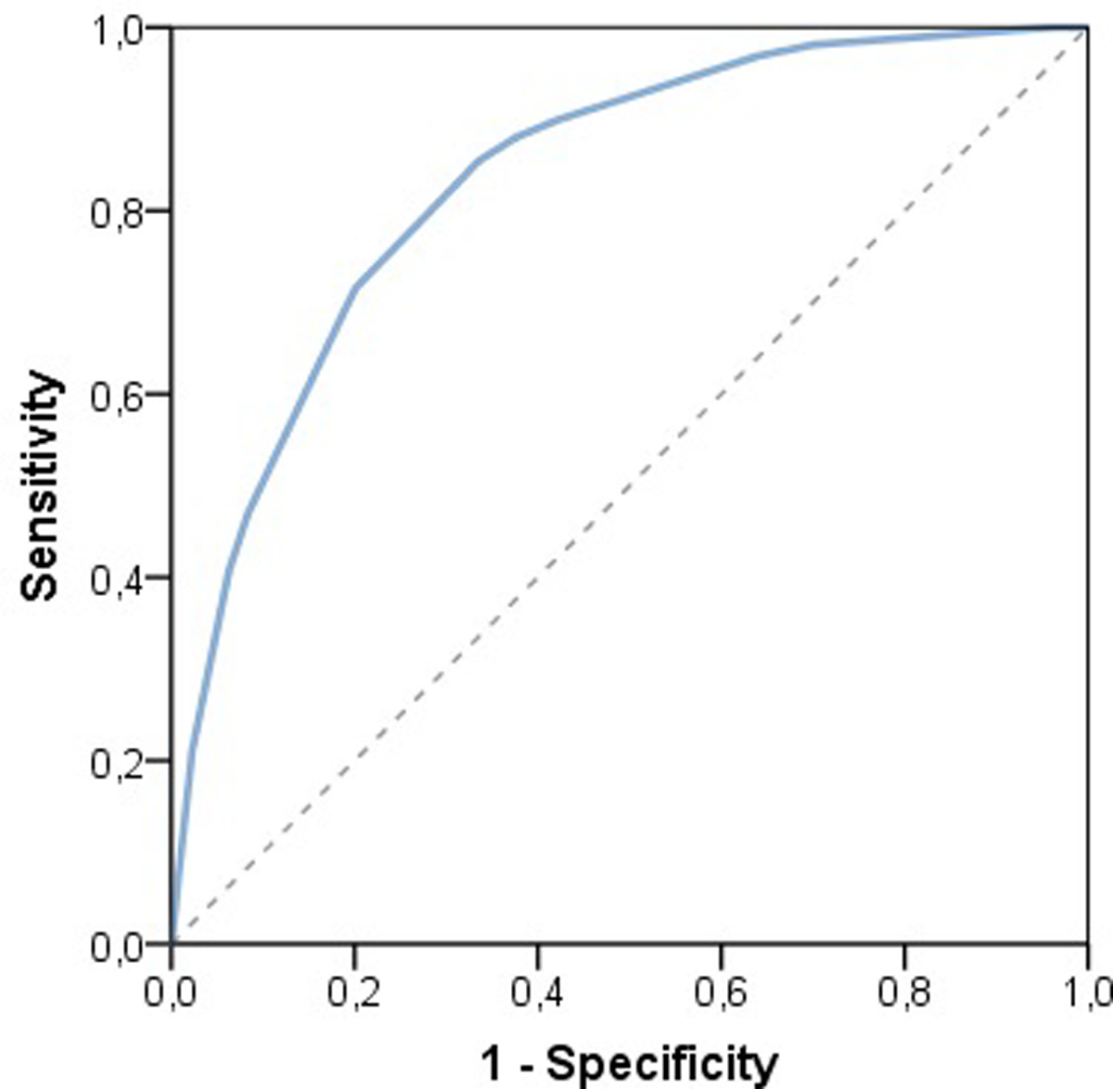
ROC curve for the decision-tree model.

The ROC curve for the decision-tree model is presented in Fig 2. The decision-tree model presented good predictive accuracy, with AUC = .84 (95% CI = .80/.87).

## Discussion

The decision-tree emerging from this study can be considered an innovative generic risk assessment tool to identify pediatric patients at greater risk of developing borderline/clinically significant psychological symptoms based on the DISABKIDS-10 Index as a screening HrQoL measure and a brief questionnaire assessing patients’ and families’ sociodemographic and clinical characteristics. The decision-tree model presented adequate validity and accuracy and allowed the identification of two high-risk (children/adolescents with asthma or diabetes and HrQoL scores below the threshold of 70.0; patients with epilepsy, cerebral palsy or obesity and HrQoL scores below 57.5) and two medium-risk profiles (children/adolescents with epilepsy, cerebral palsy or obesity and HrQoL between 57.5–80.0; children/adolescents with epilepsy, cerebral palsy or obesity, HrQoL between 80.0–87.5 and taking medication) that predicted 71.5% of borderline/clinical cases. Thus, these four groups of patients must be given priority regarding referral to specialized psychological assessment and interventions in healthcare settings.

In the total sample, 21.5% of children and adolescents presented borderline/clinically significant psychological problems according to the SDQ cut-off values. This prevalence of patient-reported psychological problems is comparable to population norms from several European countries [48–50]. However, the prevalence of borderline/clinical psychological problems differed between diagnostic groups, with patients with epilepsy or obesity presenting significantly more symptoms than those with asthma or diabetes. In addition, patients with cerebral palsy or obesity had lower HrQoL scores than children and adolescents with asthma. Previous research has shown that children and adolescents with neurological disorders are more likely to have impaired HrQoL and more psychopathological problems compared with patients with other conditions with no impairment to the central nervous system [5,51]. Recent studies have also documented poorer HrQoL and higher levels of depressive symptoms in overweight children and adolescents compared with normal-weight peers [52] or patients with other chronic conditions [53]. These findings may be explained by the weight self-stigma, victimization by peers, weight-related teasing and social marginalization that is often experienced by children and adolescents with obesity and that is reflected in their self-assessments of generic HrQoL as a multidimensional construct that includes social functioning and well-being.

The CHAID algorithm clustered the group of children and adolescents with neurological disorders and obesity into the same node, in which 27.2% of patients had borderline/clinically significant psychological problems, in contrast with a prevalence of 16.3% in the asthma/diabetes node. The results indicated that even after controlling for diagnosis, the best predictor of borderline/clinical psychological problems was the child-reported HrQoL. However, the classification-tree model defined different cut-off points for the DISABKIDS-10 scores depending on the diagnostic group: children and adolescents with asthma or diabetes are at a greater risk of borderline/clinical psychological problems if they had HrQoL scores below 70.0, whereas patients with epilepsy, cerebral palsy or obesity were at high risk if they presented HrQoL scores below 57.5 and medium risk if they had HrQoL scores between 57.5–80.0. Patients with epilepsy, cerebral palsy or obesity and HrQoL scores between 80.0–87.5 were also at medium risk if they were taking medication. These cut-off points reflect the well-documented differences in HrQoL mean scores across chronic health conditions [3,53] because neurological conditions and obesity are expected to have severe detrimental effects on HrQoL; thus, only very low HrQoL scores would be indicative of greater risk for comorbid psychological problems.

Consistent with previous studies [5,18,19], SES levels, family structure and patients’ age group were included as significant risk factors in the decision-tree model. However, although the CHAID algorithm subdivided the group of patients with asthma or diabetes and HrQoL scores above 70.0 based on significant differences between the sociodemographic groups, the proportion of borderline/clinical cases predicted by the generated profiles resembled those reported for normative samples. Male gender has been associated with higher levels of externalizing problems but not internalizing problems [5]. Thus, it was automatically excluded as a risk factor in our decision-tree model because we considered psychological problems in general without differentiating between internalizing and externalizing symptoms.

Our findings should be interpreted with caution due to some limitations in the study’s design and procedures. First, the study’s cross-sectional design precluded the establishment of causal links among variables. Thus, we cannot claim that lower HrQoL levels predict an increased likelihood of developing borderline/clinically significant psychological problems. Instead, we suggest that HrQoL and psychological problems should be considered two indicators of the broader construct of psychosocial adaptation that covary [14]. Prospective studies are needed to test whether clinically significant changes in HrQoL would result in enhanced mental health. The second limitation was the non-probabilistic sample collection method and the consequent heterogeneous distribution of patients’ and parents’ sociodemographic and clinical characteristics. Specifically, the low percentage of participants with high SES and SDQ scores within the clinical range required the dichotomization of these variables; in addition, the disease severity markers were assessed with condition-specific scales and subsequently dichotomized, which increased the intragroup variability. A third limitation involves the use of the SDQ Total Difficulties score. Although there are recent recommendations for classifying SDQ scores into internalizing and externalizing problems in low-risk samples [54], and there is empirical evidence that specific diagnostics are differentially associated with emotional or behavioral problems [5], cut-off points to differentiate SDQ scores into normal, borderline and clinical internalizing and externalizing problems are unavailable. Fourth, the small sample size for each clinical group prevented the use of stratified analyses for specific diagnosis, because the CHAID algorithm uses multiway splits, and thus it needs rather large sample sizes to ensure accuracy. Fifth, other risk factors, such as parents’ education, number of children/siblings in the family, family routines and parents’ psychological symptoms and quality of life, were not included in the present study because the predictive accuracy of the CHAID algorithm is highly dependent of the sample size and number of predictors, and should be considered in further research with larger samples of children/adolescents with chronic health conditions. Sixth, this study relied solely on child-reported data and excluded parent-reports, although parents may be more prone to report worse HrQoL and more psychological problems than children and adolescents are [55]. Finally, the DISABKIDS-10 Index is a generic cross-cultural HrQoL instrument, but only Portuguese children and adolescents with asthma, cerebral palsy, type-1 diabetes, epilepsy or overweight/obesity were included. Thus, the established cut-off points for interpreting HrQoL might be sample dependent. In addition, condition-specific instruments may be more sensitive to specific aspects of functioning that may be impacted directly by the condition and/or its treatments [56]. Future research should replicate this classification-tree model in larger samples of patients from different countries and cultural backgrounds and with other specific chronic health conditions (e.g., pediatric cancer, juvenile rheumatoid arthritis, dermatitis, etc.) using both patient- and parent-reported data and combining generic and condition-specific instruments as screeners to establish the cross-cultural validity of HrQoL cut-off points and to maximize the sensitivity of the risk assessment tool. Further studies should also consider the examination of stratified classification-tree models according to specific diagnosis, including condition-specific HrQoL instruments as screeners as well as specific indicators of disease severity and/or control of symptoms.

Despite these limitations, this is the first study to examine the interplay among clinical, sociodemographic and HrQoL data to predict the risk of developing borderline/clinically significant psychological problems, facilitating the development of a screening tool for healthcare providers. There is a growing need to identify at-risk populations in healthcare services, but the limitations of commonly used statistical methods [57] and financial/human resource constraints have made this difficult. Specifically, logistic regression analyses have been traditionally used to determine the average effect of risk factors on the likelihood of developing borderline/clinical psychological problems and, thus, they were geared toward the average member of the population, with little sensitivity to potential differences between population subgroups [58]. In contrast, the classification-tree analysis is a nonparametric technique that can be used without constraints regarding the functional form of the data [57] and that accounts for interaction effects between predictors (i.e., it allows the identification of variables that may have a predictive value only for specific clusters of pediatric patients). The major advantage of using decision-tree modeling in our study was its ability to identify high-risk subgroups of pediatric patients through the segmentation of populations into meaningful and mutually exclusive subgroups whose members share similar clinical and sociodemographic characteristics that are associated with psychological problems. Decision-trees are visual, structured/hierarchized and easy to understand; these are requirements for routine monitoring and screening in healthcare settings. In addition, the variables that emerged as predictors of borderline/clinical cases are practical for use in a risk assessment tool because brief patient-report HrQoL measures and sociodemographic and clinical data can be easily and promptly obtained by any healthcare provider.

In clinical practice, this decision-tree approach would consist of a series of questions that would follow various iterations as necessary, similar to many common diagnostic tools. According to our decision-tree model, the specific diagnostic would determine the influence of all other variables and must be considered first, followed by HrQoL assessment. Another major practical implication of this study is the establishment of condition-specific, clinically meaningful, cut-off points to interpret HrQoL data as assessed by the chronic-generic DISABKIDS-10 Index, which is an essential measurement property when using HrQoL instruments in clinical decision-making [25]. This cost-effective generic risk assessment tool may contribute to overcoming barriers to psychosocial screening and monitoring and may become a useful tool to identify at-risk children and adolescents. For children and adolescents classified into low-risk profiles (less than 25% odds of presenting borderline/clinical psychological symptoms) clinical management should be based on other factors. However, the classification of a pediatric patient into a medium- or high-risk profile (25%-50% and more than 50% odds of presenting borderline/clinical psychological symptoms, respectively) should have a substantial impact on the clinical management and allocation of health resources, particularly with regard to referral for specialized psychological assessment and intervention. In addition to allowing healthcare providers to easily identify children and adolescents who are most likely to present borderline/clinically significant psychological problems, our findings suggest that psychosocial interventions aimed at improving generic physical, psychological and social functioning may be effective in promoting the mental health of pediatric patients.

## Supporting information

S1 File Data set (*N* = 736). (XLSX)
